# Plasma-Induced Crystallization of TiO_2_ Nanotubes

**DOI:** 10.3390/ma12040626

**Published:** 2019-02-20

**Authors:** Metka Benčina, Ita Junkar, Rok Zaplotnik, Matjaz Valant, Aleš Iglič, Miran Mozetič

**Affiliations:** 1 Department of Surface Engineering and Optoelectronics, Jožef Stefan Institute, Jamova 39, Ljubljana 1000, Slovenia; metka.bencina@ijs.si (M.B.); rok.zaplotnik@ijs.si (R.Z.); miran.mozetic@ijs.si (M.M.); 2 Materials Research Laboratory, University of Nova Gorica, Vipavska 13, Nova Gorica 5000, Slovenia; matjaz.valant@ung.si; 3 Institute of Fundamental and Frontier Sciences, University of Electronic Science and Technology of China, Chengdu 610054, China; 4 Laboratory of Biophysics, Faculty of Electrical Engineering, University of Ljubljana, Tržaška 25, Ljubljana 1000, Slovenia; ales.iglic@fe.uni-lj.si

**Keywords:** TiO_2_ nanotubes, crystallization, gaseous plasma, biological response

## Abstract

Facile crystallization of titanium oxide (TiO_2_) nanotubes (NTs), synthesized by electrochemical anodization, with low pressure non-thermal oxygen plasma is reported. The influence of plasma processing conditions on TiO_2_ NTs crystal structure and morphology was examined by X-ray diffraction (XRD) and scanning electron microscopy (SEM). For the first time we report the transition of amorphous TiO_2_ NTs to anatase and rutile crystal structures upon treatment with highly reactive oxygen plasma. This crystallization process has a strong advantage over the conventional heat treatments as it enables rapid crystallization of the surface. Thus the crystalline structure of NTs is obtained in a few seconds of treatment and it does not disrupt the NTs’ morphology. Such a crystallization approach is especially suitable for medical applications in which stable crystallized nanotubular morphology is desired. The last part of the study thus deals with in vitro biological response of whole blood to the TiO_2_ NTs. The results indicate that application of such surfaces for blood connecting devices is prospective, as practically no platelet adhesion or activation on crystallized TiO_2_ NTs surfaces was observed.

## 1. Introduction

Self-aligned TiO_2_ nanotubes synthesized by electrochemical anodization of Ti foil are highly promising materials for biomedical applications due to their advanced biocompatibility in comparison to commonly used plain metal surfaces, and as such can be employed as orthopaedic and dental implants, vascular stents, antibacterial devices or surfaces and smart drug-delivery platforms [1,2,3,4,5,6,7,8]. Improved biological response to the TiO_2_ NTs is, among others, the result of increased surface roughness and larger surface area. Moreover, the tunable morphology of NTs that can be controlled by altering the anodization parameters such as voltage and time, allows for selective response of biological material. For instance, proteins, platelets and cell adhesion, cell morphology, proliferation and differentiation are highly affected by the TiO_2_ NTs diameter [9]. This is especially important in applications, in which the adhesion of certain cell types is preferred, as in the case of vascular stents. 

As-anodized amorphous TiO_2_ NTs are, therefore, often subjected to crystallization process in order to enhance their bio-performance. It has been shown that annealed TiO_2_ NTs layers with anatase crystal structure have a much better corrosion resistance and bioactivity than amorphous NTs; moreover, they tend to induce more, and faster, hydroxyapatite deposition, which is crucial for successful bone bonding (osteointegration) ability of the body implants [10,11,12]. In addition, the proliferation and mineralization of osteoblasts cultured on anatase or a mixture of anatase⁄rutile TiO_2_ NTs was significantly higher than on the surface of as-anodized amorphous TiO_2_ NTs [13]. Crystallized TiO_2_ NTs have also good bacterial resistance; it was found that such layers inhibit the growth of *S. aureus* and *P. aeruginosa* [5], which is important for designing medical devices, since implant-associated infections present a serious health care concern. In addition, the enhanced biocompatibility of crystalline TiO_2_ NTs could also be linked with increasing Ti-OH functional groups, responsible for enhancing wettability and apatite deposition [14,15] and increasing stability in bioliquids [16]. 

Crystallization of TiO_2_ NTs induced by annealing in a conventional furnace is a well-established process; the atom rearrangements occur due to elevated temperatures. However, such crystallization requires high temperatures and is time consuming in comparison with the plasma-induced crystallization presented in this contribution. For instance, in order to achieve anatase, mixture of anatase/rutile and rutile crystal structure, TiO_2_ NTs should undergo annealing in the furnace for at least 2 h at 450 °C, 550 °C [10] and 800 °C [17], respectively. Besides, changes in the morphology of TiO_2_ NTs present a significant drawback of such crystallization process; the annealing in a furnace at 800 °C results in the transition of anatase NTs to a dense rutile layer [17]. By contrast, hydrothermal treatment presents an intriguing low-temperature crystallization method that is catalysed by pressure and mineralizing agents [18]. Liu et al. [19] reported hydrothermal reaction in an autoclave at a temperature of less than 180 °C for 4 h, which led to the amorphous-to-ananase transition of TiO_2_ NTs. In spite of low temperature requirements, hydrothermal crystallization adversely alters TiO_2_ NTs morphology and leads to the formation of aggregated anatase nano-particles on the surface of NTs at about 200 °C [20]. Lamberti et al. [14] demonstrated another attractive near-room temperature (50 °C) crystallization process of amorphous TiO_2_ NTs, which led to anatase crystal phase formation after only 30 min of sample exposure to water vapour [14,20]. However, such a crystallization method also initiates the formation of crystals at the outer and inner walls of the NTs, which finally leads to transformation of nanotubes to nanorods. For this reason, there is an indispensable need to develop novel crystallization processes that would preserve the morphology of the materials.

In the present research, the transformation of rather amorphous TiO_2_ NTs to anatase or mixture of anatase/rutile crystal structure by a non-thermal oxygen plasma process, is, according to our best knowledge, reported for the first time. Fast amorphous-to-anatase or -anatase/rutile transition and unaltered NTs morphology are the major advantages of such crystallization before conventional processes, for which the preservation of morphology is still a challenge. Although such a time-saving method is convenient for inducing crystallinity of the nanomaterials/thin films [21,22,23], there is a lack of information about the mechanisms involved in the crystallization process. In this contribution, the influence of plasma processing conditions on the crystal structure and morphology of the TiO_2_ NTs is therefore examined. In addition, the biological aspects of plasma treated TiO_2_ NTs are presented. It has already been shown that surfaces treated by highly reactive oxygen plasma improve biological response. For instance, Junkar et al. [24] showed that plasma treatment allows for TiO_2_ NTs surface functionalization and enhancement of osteoblast-like cell responses. Authors also confirmed cleaning and sterilizing effects of plasma treatment [25]. Moreover, the wettability of ZrO_2_ was noticeably improved by an oxygen plasma treatment, which promoted the attachment, proliferation and differentiation of human osteoblast-like cells [26]. Therefore, simultaneous plasma treatment and crystallization of the material with already tunable morphology offers highly needed improvements for devices used in biomedical applications.

## 2. Materials and Methods 

### 2.1. Materials

Titanium foil (Advent, 0.1 mm thickness, 99.6%), ethylene glycol (Fluka, ≥99.5%), ammonium fluoride—NH_4_F (Sigma Aldrich, 28.0–30.0%), hydrofluoric acid—HF (Sigma Aldrich, ≥40%) acetone (Honeywell Riedel–de Haen, 99.5%), ethanol (Sigma Aldrich, 96%), phosphate-buffered saline—PBS (Sigma Aldrich), and glutaraldehyde solution (Sigma Aldrich, 25% in H_2_O) deionized water (miliQ).

### 2.2. Synthesis of TiO_2_ NTs by Electrochemical Anodization

The TiO_2_ NTs were fabricated by an electrochemical anodization method as described in Refs. [24,25]. The synthesis was carried out at room temperature (≈20 °C) in a two electrode system (Pt/Ti) with the size 10 × 10 mm^2^ and working distance of 15 mm. The thickness of the Ti electrode was 0.10 mm. Before anodization, the Ti foil was ultrasonically cleaned in acetone, ethanol and deionized water for 5 min in each and further dried under a nitrogen stream. The electrolyte used in this step was composed of ethylene glycol and NH_4_F (0.35 wt.%) and H_2_O (1.7 wt.%). The nanotubular layer grown in this step was then detached from the substrate with a successive ultrasonication in H_2_O, acetone and ethanol in order to obtain a pre-dimpled surface, which led to enhanced homogeneity of the NTs’ surface. In the second step of anodization process, the pre-treated Ti foils were used as a substrate to grow NTs. The electrolyte based on the ethylene glycol, containing water and HF was used and the detailed synthesis parameters of these steps are presented in Table 1. The as-synthesized NTs were kept in ethanol for 2 h in order to remove components from the electrolyte and then dried under a nitrogen stream. The as-prepared NTs were used for further plasma processing (output power 200–800 W for different times) or annealed at 450 °C for 2 h in a furnace with annealing/cooling rate 8 °C/min.

### 2.3. Oxygen Plasma Treatment

Treatment of TiO_2_ NTs was performed by oxygen plasma in the plasma reactor designed in house. The system was evacuated with a two-stage oil rotary pump with a nominal pumping speed of 80 m^3^ h^−1^. The discharge chamber was a Pyrex tube with a length of 80 cm and an inner diameter of 3.6 cm. Gaseous plasma was created with an inductively coupled radiofrequency (RF) generator (CESAR 1310, Advanced Energy, Fort Collins, Colorado, USA), operating at a frequency of 13.56 MHz and a nominal power of 1000 W. Generator powers between 200–800 W were used. Commercially available oxygen was leaked into the discharge chamber and the pressure was measured with an absolute vacuum gauge. The pressure during the plasma treatment was fixed at 50 Pa which allows for the highest degree of dissociation of oxygen molecules for this particular plasma reactor. The samples were placed on an object glass and treated for different periods of time and with different output powers, one at a time. Details about the behaviour of reactive gaseous species versus nominal power of the RF generator are presented elsewhere [27].

### 2.4. Characterization

#### 2.4.1. X-ray Diffraction (XRD) Spectroscopy Analysis

X-ray diffraction (XRD) was performed using MiniFlex 600 Benchtop X-ray diffractometer (Rigaku, Tokyo, Japan) equipped with Cu K-α radiation (1.541 Å) over the 2θ range 10–70°, with a step size of 0.017°, divergence slit of 0.218° and counting step time of 25 s in continuous scanning mode. Carbon tape was used to mount the samples on the glass holder. 

#### 2.4.2. Scanning Electron Microscope (SEM) Analysis

Morphology of the materials was analysed with a JSM 7100F scanning electron microscope (SEM, JEOL Ltd., Tokyo, Japan). For biological evaluation of platelets on the surface, the samples were coated with gold/palladium and examined by SEM at an accelerating voltage of 15 kV. The test was done in triplicates and only representative images are shown in this paper.

#### 2.4.3. Temperature Measurements

During the exposure of the samples to plasma, the temperature was measured with a custom-made K-type thermocouple). Chromel and alumel wires (Goodfellow Cambridge Ltd., Huntingdon, UK) were spot welded on the rear of the samples so they were not influenced by plasma. A Keithley Model 2100 digital multimeter (Keithley Instruments/Tektronix, OH, USA) and custom-made software were used to record the time evolution of the samples temperature. 

### 2.5. In Vitro Biological Response—Interaction With Whole Blood 

All subjects gave their informed consent for inclusion before they participated in the study. The study was conducted in accordance with the Declaration of Helsinki, and the protocol was approved by the Ethics Committee of Slovenia (56/03/10). Whole blood was obtained from healthy volunteers via vein puncture. The blood was drawn into 9 ml tubes already coated with trisodium citrate anticoagulant. The material samples—Ti foil, amorphous and annealed (anatase) TiO_2_ NTs of 100 nm in diameter (7 × 7 mm^2^)—were incubated with the 250 μL of whole blood for 45 min at room temperature in the 24-well cell culture plates. Afterwards, 250 μL of PBS was added to the incubated samples. The blood with PBS was then removed and the samples were rinsed 3 times with 250 μL of PBS in order to remove weakly adherent platelets and other biological material. The adherent platelets were further fixed by 400 μL of 0.5% glutaraldehyde solution for 2 h at room temperature. Then the materials were rinsed with PBS and dehydrated by using a graded ethanol series (50 vol.%, 70 vol.%, 80 vol.%, 90 vol.%, 100 vol.% and again 100 vol.% of ethanol) for 5 min and in the last stage (100 vol.% ethanol) for 10 min. Afterwards the samples were dried with liquid nitrogen and stored in vacuum. 

## 3. Results and Discussion

### 3.1. Crystal Structure Analysis

**Figure 1:**
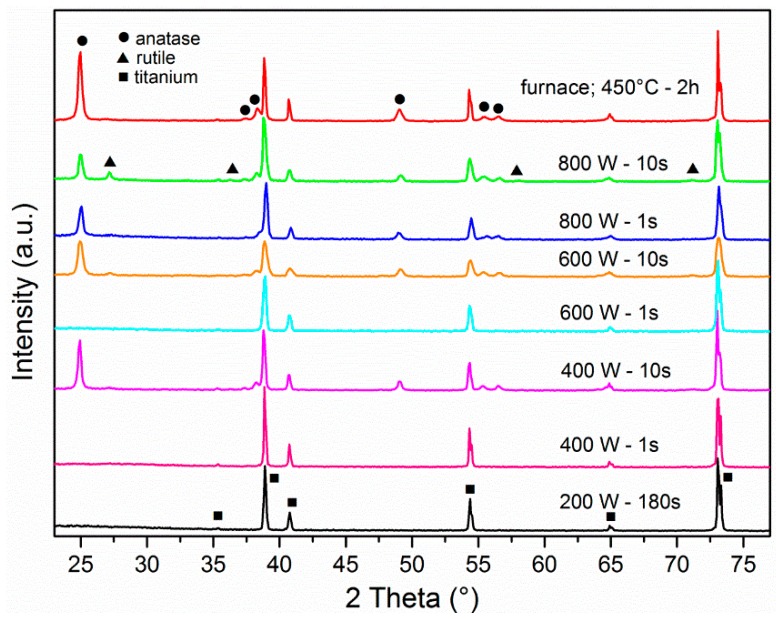
X-ray diffraction (XRD) patterns of TiO_2_ NTs of 100 nm diameter measured after plasma treatment at different powers; 200, 400, 600 and 800 W for different times and TiO_2_ NTs of 100 nm diameter after annealing in a furnace at 450 °C for 2 h.

**Figure 2:**
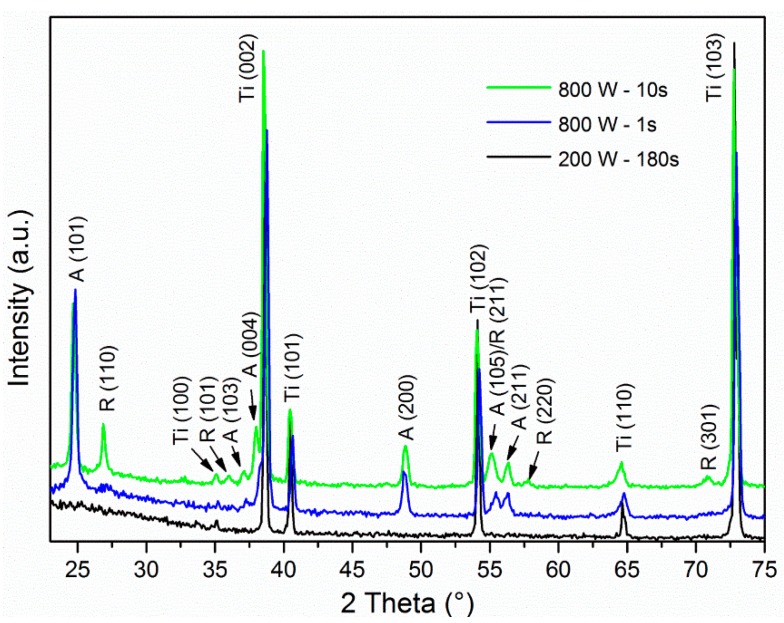
Magnified X-ray diffraction (XRD) patterns of TiO_2_ NTs of 100 nm diameter measured after plasma treatment at different powers; 200 and 800 W for different times, in order to show the difference between amorphous, anatase and rutile crystal phase of TiO_2_ NTs. A = characteristic XRD peaks for anatase crystal structure, R = characteristic XRD peaks for rutile crystal structure and Ti = characteristic XRD peaks for Ti foil.

The TiO_2_ NTs were subjected either to annealing or highly reactive oxygen plasma treatment at different input powers and at different treatment times and the influence of such treatments was further studied in terms of altered surface morphology and crystallization. It has been shown in our previous work [25] and recently also in Ref. [28] that TiO_2_ NTs were deformed after exposure to elevated temperatures and that NTs’ stability depended on diameter (larger diameter NTs are more resistant to thermal degradation). In the present study, the alteration in crystallinity of TiO_2_ NTs was studied by X-ray diffraction spectroscopy, which revealed that the treatment with highly reactive oxygen plasma at different powers significantly influences the TiO_2_ NTs crystal structure. The as-anodized TiO_2_ NTs samples are amorphous and remain uncrystallized even after 3 min of exposure to highly reactive oxygen plasma at 200 W (Figure 1). The XRD data show that the Ti foil, which is used as a substrate for TiO_2_ NTs fabrication, is detected by XRD although NTs of 100 nm in diameter have a length of about 2.5 µm. The higher power of plasma, more than 400 W, seems to already induce crystallization if samples are exposed to plasma for more than 10 s. Actually a mixture of anatase and rutile crystal phase (hardy observable peak at 2θ = 36.5° as seen in Figure 1) appears after the treatment of TiO_2_ NTs for 10 s in plasma with the power of 400 W and 600 W. Interestingly, the 1 s plasma exposure at these conditions is not enough to initiate the change of crystal structure, since no anatase nor rutile peaks were detected. However, the plasma treatment at 800 W for 1 s and 10 s results in anatase and a mixture of anatase/rutile crystal phases, respectively (Figure 1 and Figure 2). For comparison, the annealing of TiO_2_ NTs in a conventional furnace at 450 °C requires 2 h to induce the transition of amorphous phase to anatase crystal structure.

**Figure 3:**
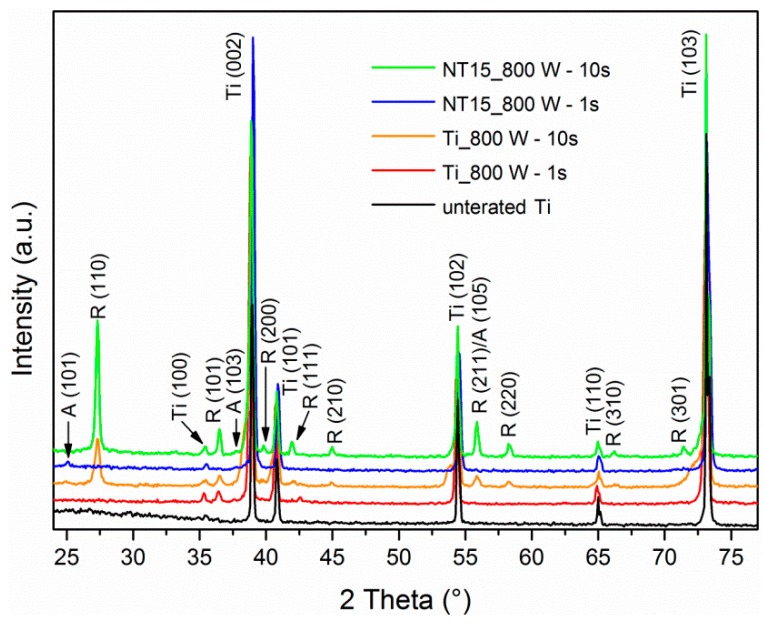
X-ray diffraction (XRD) patterns of untreated Ti foil and Ti foil and TiO_2_ NTs of diameter 15 nm (NT15) measured after plasma treatment at 800 W for 1 s and 10 s. A = characteristic XRD peaks for anatase crystal structure, R = characteristic XRD peaks for rutile crystal structure and Ti = characteristic XRD peaks for Ti foil.

In order to study the effect of synthesized TiO_2_ layer thickness on the crystallization in plasma, TiO_2_ NTs of 15 nm in diameter and length of about 0.2 µm were treated with oxygen plasma at 800 W for 1 s and 10 s. Only one anatase-phase peak at 2θ = 25° (101) was detected for the TiO_2_ NTs sample treated with plasma at 800 W for 1 s, indicating that the sample is still mainly amorphous (Figure 3). Interestingly, for TiO_2_ NTs of 100 nm in diameter the anatase crystal phase is already observed after 1 s of treatment at 800 W (Figure 1 and Figure 2). However, the rutile crystal phase without any evidence of anatase prevails when plasma treatment at 800 W is prolonged to 10 s for TiO_2_ NTs of 15 nm in diameter (Figure 3). For comparison, TiO_2_ NTs of 100 nm in diameter crystallize in mixture of anatase/rutile phase after prolonged plasma treatment (10 s). The XRD spectra of plain Ti foil, which is used as a substrate for TiO_2_ NTs fabrication, are presented in Figure 3; it can be observed that XRD pattern of the plasma-treated sample at 800 W for 1 s exhibit small rutile and anatase peaks at 2θ = 36.5° and 37.5°, respectively. However, an intense rutile-phase peak at 2θ = 27° is confirmed after the exposure of the Ti foil to plasma of power 800 W for 10 s. These results show that although smaller-sized nanoparticles require less intensive plasma conditions to induce crystallization [21,29], the thermal effects on TiO_2_ NTs with 2.5 µm length and 0.21 µm length may be different. It is also noteworthy that plasma treatment induce the formation of crystalline oxide layer on the surface of the Ti foil. 

### 3.2. Morphology Analysis

**Figure 4:**
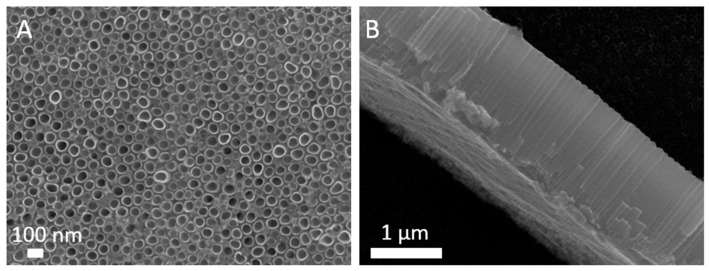
Scanning electron microscope (SEM) images of untreated as-anodized TiO_2_ NTs with diameter of 100 nm; (**A**) top view, (**B**) side view.

**Figure 5:**
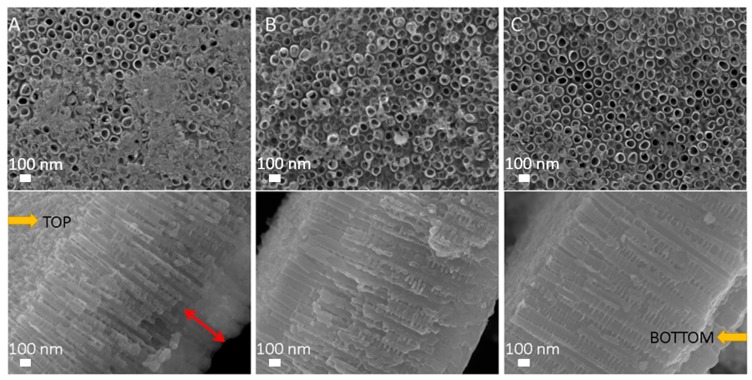
SEM images (above: top view and below: cross sectional view) of TiO_2_ NTs of 100 nm in diameter treated with oxygen plasma at (**A**) 800 W for 10 s, (**B**) 800 W for 1 s and (**C**) 400 W for 10 s. The red arrow indicates destroyed bottom layer of NTs.

The morphology of as-anodized TiO_2_ NTs (100 nm in diameter) is shown in Figure 4, while the morphology of TiO_2_ NTs exposed to oxygen plasma is shown in Figure 5. It is noteworthy that TiO_2_ NTs formed on the Ti foil are stable at plasma conditions used in the present study, except the NTs treated with plasma of power 800 W for 10 s. SEM analysis of TiO_2_ NTs treated at 800 W for 10 s showed that the NTs structure is destroyed as can be seen from Figure 5, where the comparisons between plasma-treated samples at 800 W for 10 s, 800 W for 1 s and 400 W for 10 s are presented. The tops of TiO_2_ NTs treated at 800 W for 10 s are partially closed, most probably due to collapsing of NTs walls (Figure 5A) and increase in stoichiometric oxide on the surface. It is also evident form Figure 5A that the bottom layer of the NTs is destroyed or even that there is a formation of oxide layer as already observed in Ref. [30], which could be caused by a longer exposure to high temperatures. Mazare et al. [5] observed the same loss of nanotubular structure after annealing at high temperatures (i.e., 550 °C, 650 °C and 750 °C). It was previously reported that the formation of a rutile oxide layer is initiated at the metal-nanotube interface during the annealing [30], and therefore changes in crystal structure firstly occur at the bottom layer of the NTs. A similar structure, although not destroyed, was formed when the TiO_2_ NTs were treated at 800 W for 1 s (Figure 5B). However, the morphology of TiO_2_ NTs treated at 400 W for 10 s is not destroyed and the tubes are well defined and opened (Figure 5C). 

**Figure 6:**
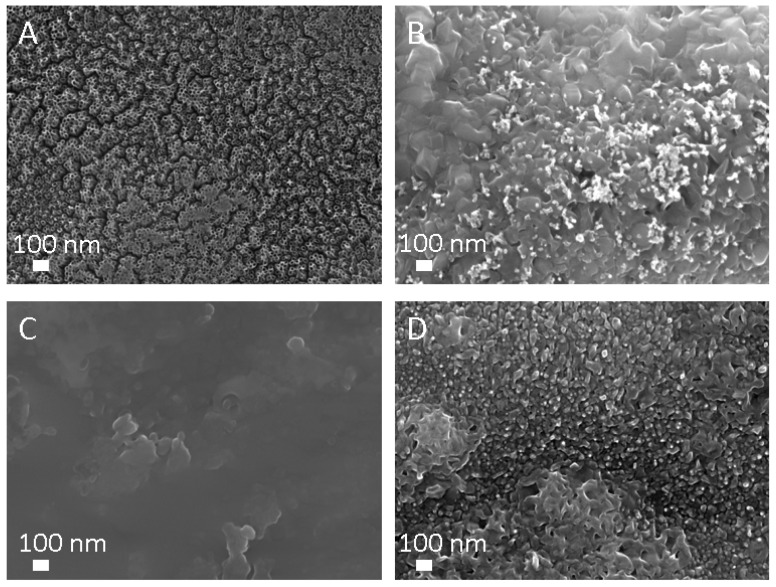
SEM images (top view) of TiO_2_ NTs of 15 nm in diameter after plasma treatment at (**A**) 800 W for 1 s, (**B**) 800 W for 10 s and Ti foil after plasma treatment at (**C**) 800 W for 1s and (**D**) 800 W for 10 s.

SEM analysis revealed that TiO_2_ NTs of 15 nm in diameter have open tops and their structure is not destroyed after treatment with plasma at 800 W for 1 s (Figure 6A). However, NTs are completely destroyed after plasma treatment at 800 W for 10 s (Figure 6B). Ti foil morphology is unaltered after 1 s plasma treatment at 800 W (Figure 6C), while changes in the morphology of Ti foil after plasma treatment at 800 W for 10 s can be observed; the surface is covered with nanostructures (fused oxide particles) as seen in Figure 6D.

### 3.3. Temperature Measurements

Crystallization of the materials in a furnace is governed by the change in temperature. Several studies report on light-induced [31,32,33], laser-induced [34,35], and microwave-induced [36] crystallization, which are also correlated with sample heating. In the present study we used a plasma treatment and the temperature of the samples during the treatments in correlation with plasma output power were measured. As evident from Table 2, the temperature of the sample increases with increasing output power of the plasma reactor in the time range up to about 10 s. It has been previously reported that plasma used in the present study operates in two different modes, E and H; The E-mode prevails at a low input power and is defined by a relatively low electron density, high electron temperature and low light emission, while the H-mode at higher input power represents a higher electron density, somehow lower electron temperature and higher light emission [37]. Zaplotnik et al. [27] measured the transition from E- to H–mode in the same plasma system as used in this study. It was found that the E–H transition occurs at the generator power of about 400 W at the pressure of 50 Pa. Below the output power of 400 W, the power transmission to plasma is not ideal; for example, at generator forward power of 200 W, the forward minus reflected power is about 65 W, while for example at 400 W, there is much lower reflected power which means the transmission of power is much better. Exposure of TiO_2_ NTs to H-mode plasma subjects the sample, among others, to more intense electron density and light emission. Therefore, the samples exposed to H-mode for a sufficient time could crystallize in seconds, while exposure of samples to E-mode, even for 3 min, does not initiate the crystallization. 

Although the process of nanoparticles synthesis by plasma treatment has already been reported [21], there is a lack of reports and detailed mechanisms about the crystallization upon treatment with non-thermal plasmas. Ohsaki et al. [23] claim that the crystallization of sol-gel derived TiO_2_ thin films can be achieved within a few minutes by a non-thermal plasma processing. The authors suggest that crystallization of the materials should be derived from the excitation by the RF electromagnetic field and not plasma itself. Similarly, An et al. [38] speculate that formation of the crystalline grains of BaTiO_3_ is due to the energy provided by ion bombardment. Contrary, Kramer et al. [21] suggested that the silicon nanoparticles exceed the gas temperature in non-thermal plasmas (370–430 K/97–157 °C) to the point of sufficient temperature for crystallization (up to 700 K/427 °C), although measurements showed that the average temperature of the silicon nanoparticles exposed to plasma was close to the gas temperature. According to the authors [21], crystallization of the nanoparticles by non-thermal plasma occurs due to the electron ion recombination and reactions of radicals on the nanoparticle’s surface. Similarly, Lopez et al. [22] demonstrated that plasma exposure allowed for crystallization of silicon nanoparticles due to the heating of the nanoparticles to the temperature of 1100 K/827 °C. 

In present study, the correlations between TiO_2_ NTs crystal structure and plasma processing conditions have been studied. It has been shown that TiO_2_ NTs crystallize to anatase and at appropriate conditions also to rutile phase when exposed to highly reactive oxygen plasma for sufficient time and at appropriate plasma power. It was shown that the mixture of anatase/rutile crystal structure was achieved after 10 s at the power of 400 W, where the measured temperature of the sample was above 1210 °C. These operating conditions allow more intense physical processes which occur during interaction of plasma species with the sample. We presume that these interactions contribute to the alteration of the sample’s crystal structure, mainly due to rapid heating of the sample induced by surface recombination of neutral oxygen atoms and ion neutralization. However, the contribution of these two mechanisms to the floating sample heating in electrodes low pressure oxygen plasma are not of the same order. The densities of the ions are a few orders of magnitude lower than the densities of the neutral oxygen atoms and, therefore, the heating of the sample due to the ion neutralization can be neglected [39]. Therefore, the heating of samples (*P_H_*) can be expressed with the formula (1):

*P_H_ = γ·j·W_D_*/2, (1)

where *γ* is the recombination coefficient of the oxygen atoms for the specific surface, *j* is the flux of the oxygen atoms onto the sample surface and *W_D_* is the dissociation energy of oxygen molecules. At the same time the sample is being cooled, where two most important mechanisms are radiation and convection. The radiation part (*P_r_*) is described with the Stefan–Boltzmann law (2):

*P_r_ = A·ε·σ·T*^4^, (2)

where *A* is the surface area of the sample, ε is the emissivity, σ is the Stefan–Boltzmann constant and *T* is the sample temperature. The convection part (*P_c_*) is a linear function of temperature and can be described as (3):

*P_c_ = h·A·T*, (3)

where *h* is the convection coefficient. The sample immersed in plasma reaches steady temperature when the heating part is equal to the sum of the cooling parts (4):

*P_H_ = P_r_ + P_c_*, (4)

**Figure 7:**
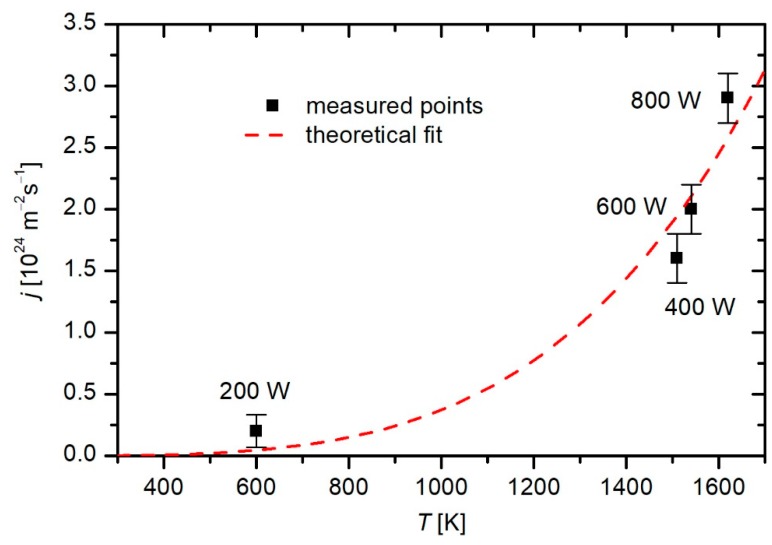
Flux of oxygen atoms and the steady temperature of the NTs samples of 100 nm in diameter.

From here we can see that larger flux of the oxygen atoms means a higher temperature of the sample. In Figure 7 the flux of oxygen atoms versus the steady temperature of the samples of NTs of 100 nm in diameter are presented. In this figure the measure points are those presented in Table 2, and the corresponding fluxes were measured with a cobalt catalytic probe. The detailed measurement technique is described elsewhere [27]. It should be noted that in this case only the measured points of samples that reached the steady temperature were used. The theoretical fit is that derived from previous formulas for cooling and heating of samples in plasma [27] taking into account the coefficient *γ* = 1. 

Destruction of the NTs was observed at higher power plasma (800 W) at prolonged treatment (more than a second). The crystallization mechanisms of TiO_2_ in plasma can be correlated with RF electromagnetic field, vacuum ultraviolet radiation, interaction with radicals and ions which induce sample heating, and chemical reactions. However, the prevailing influence of each parameter alone and its possible synergistic influence is not yet understood and definitely beyond the scope of this paper. Further studies in this direction should be conducted. The results of this study confirmed that the temperature, which was measured on the bottom surface of the samples during the treatments, plays an important role in TiO_2_ NTs crystallization. 

### 3.4. In Vitro Biological Response—Interaction with Whole Blood

**Figure 8:**
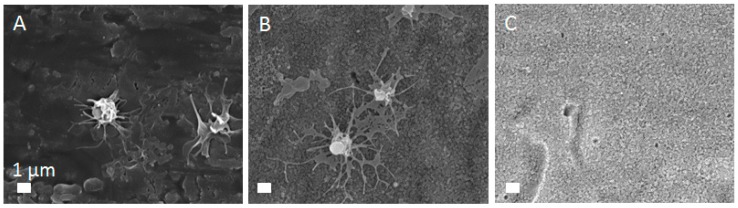
Platelets adhesion and activation on (**A**) Ti foil, (**B**) amorphous and (**C**) annealed TiO_2_ NTs with 100 nm in diameter.

The approach to rapidly alter the crystal structure of TiO_2_ NTs without influencing their morphology is an intriguing way for surface modification of NTs used in bio-applications. As mentioned before, the crystal structure of a material affects its performance in biological applications. For instance, it has been shown that platelet adhesion and activation on the crystallized TiO_2_ NTs depends of the annealing temperature and crystal phase [40]. More precisely, authors showed that a certain amount of pure anatase in the TiO_2_ NTs leads to better platelet adhesion, which is beneficial for successfully integration of dental implants. By contrast, rutile-phase TiO_2_ NTs reduced platelet adhesion and activation, perhaps due to the formation of a hydration layer, which reduced intimate contact area between a platelet and the surface [28]. This particular case is advantageous for vascular stents, for which the inhibition of adhesion and activation of platelets is preferred, since platelets’ aggregation leads to a blood clot formation and stent thrombosis. Results of platelets performance on the surface of TiO_2_ NTs of 100 nm in diameter prepared in present study are shown in Figure 8. Platelets adhere to the surface of Ti foil with filopodia, while adhesion on the amorphous TiO_2_ NTs is done via lamelopodia and fillopodia. This indicates that the amorphous TiO_2_ NTs provide a better environment for platelet adhesion and activation. By contrast, platelets could not be found on the surface of annealed TiO_2_ NTs with anatase crystal structure. No biological material was detected on these surfaces, as platelets seemed not to adhere on these surfaces. These results indicate that anatase crystal structure (obtained by plasma or annealing) of TiO_2_ NTs does not provide appropriate conditions for platelets adhesion, which is beneficial from the perspective of vascular stent applications. In previous publications we showed increased hydrophilicity and reduced electrolyte residual (fluorine) on the surface of annealed TiO_2_ NTs [26]. Since fluorine is toxic to cells, its lowered content could be the reason for the better performance of platelets. It should be also considered that besides crystal structure also surface chemistry and wettability (surface charge), as well as morphology of the material play important role in platelet response, therefore further studies of TiO_2_ NTs interactions with platelets are needed. 

## 4. Conclusions

Non-thermal oxygen plasma induced crystallization of TiO_2_ NTs synthesized by electrochemical anodization of Ti foil. The transition of the NTs’ amorphous phase to anatase and/or rutile crystal structure was obtained within a few seconds of exposure to oxygen plasma without changing the desired morphology of NTs. Although the gas temperature of non-thermal plasma was well below the thermal crystallization temperature, the sample temperature rose above 1300 °C at a certain plasma treatment condition e.g. the output power of 800 W and 10 s plasma treatment time. Results provide evidence that a rapid temperature increase of samples treated in plasma is correlated with crystallization of TiO_2_ NTs. Good correlation between the flux of oxygen atoms and increase in temperature of the sample was found; however, other parameters in plasma may also play a vital role in crystallization and should be further studied. It should be emphasized that crystallization in plasma is also influenced by the NTs diameter, as NTs with 15 nm in diameter seem to crystalize prevalently to the rutile phase, which was at our experimental conditions not the case for NTs of 100 nm in diameter. Moreover, the in vitro biological response of whole blood with crystalline TiO_2_ NTs showed that the crystallization reduces adhesion and activation of blood platelets, which is of particular interest for designing medical devices that are likely to contact blood, such as vascular stents.

## Figures and Tables

**Table 1:** Influence of synthesis conditions on the diameter and length of the TiO_2_ nanotubes (NTs).

Sample	Voltage (V)	Time (h)	Electrolyte (wt.%)	Diameter (nm)	Length (µm)
TiO_2_ NTs–15 nm	10	1	HF (1.1), H_2_O (15.3), EG	15	0.21
TiO_2_ NTs–100 nm	58	2.5	HF (1.0), H_2_O (11.0), EG	100	2.53

**Table 2:** Effect of radiofrequency (RF) forward (output) power and plasma processing time on the temperature and the crystal structure of TiO_2_ NTs of 100 nm in diameter.

Forward Power (W)	Time (s)	Temperature (°C)	Crystal Structure
200	180	300	amorphous
400	1	660	amorphous
400	10	1210	anatase + rutile
600	1	970	amorphous
600	10	1240	anatase + rutile
800	1	1030	anatase
800	10	1320	anatase + rutile
